# IL-15 Complexes Combined with PD-1 Blockade Affect Immune Cell Distribution, Localization, and Immune Signatures in Regressing Versus Non-Regressing Metastatic Breast Tumors

**DOI:** 10.3390/ijms262311490

**Published:** 2025-11-27

**Authors:** Josef W. Goldufsky, Anjelica F. Reyes, Allie A. Heller, Malia E. Leifheit, Maram N. Albalawi, Noah T. King, Timothy M. Kuzel, Jeffrey R. Schneider, Amanda L. Marzo

**Affiliations:** 1 Department of Internal Medicine, Division of Hematology and Oncology, Rush University Medical Center, Chicago, IL 60612, USAmalia_e_leifheit@rush.edu (M.E.L.);; 2 Department of Microbial Pathogens and Immunity, Rush University Medical Center, Chicago, IL 60612, USA

**Keywords:** anti-PD-1, IL-15 complexes, immunofluorescence, intratumoral, breast cancer, metabolic pathways

## Abstract

Rapid advancements in understanding how the immune system can eliminate tumors have quickly translated into breakthroughs in developing cancer therapeutics. Immune checkpoint inhibitors (ICIs) have shown great promise in several cancers; however, resistance can affect up to two-thirds of patients receiving ICIs. A significant limitation of the effectiveness of anti-PD-1 therapy centers around the insufficient levels of immune cells needed to recognize and kill cancer cells compared to the number of suppressive immune cells within the tumor microenvironment. Determining what is required to overcome the resistance to anti-PD-1 therapy in breast cancer remains a critical need. Our data demonstrate that IL-15 complexes injected intratumorally in combination with PD-1 blockade therapy induce regression of established luminal B mammary breast tumors. We show that IL-15 alone or in combination with anti-PD-1 drives changes in gene expression of pathways associated with TCR and co-stimulatory signaling, immune cell adhesion, and migration. Furthermore, we show that intratumoral injection of IL-15 complexes traffics to the tumor-draining lymph node, as evidenced by Light sheet microscopy, and colocalizes with the anti-PD-1 monoclonal antibody. We also identify the immune signatures, localization, and distribution of immune cells in regressing and non-regressing breast tumors.

## 1. Introduction

Over the last decade, new immunotherapies have revolutionized the field of immuno-oncology with the ability to harness immune responses, generating additional first-line treatment modalities alongside radiation and chemotherapeutics. Clinical trials using immune checkpoint inhibitors (ICIs) have yielded positive outcomes in many types of cancer, including breast cancer [1]. Unfortunately, complete responses to ICIs are still limited to a minority of patients, with most patients partially responding or not responding. A significant restriction in anti-tumoral immune responses that limits the efficacy of clinically available immunotherapies is the exclusion from the tumor microenvironment (TME) of cytotoxic immune cell populations within the TME. For example, the majority of breast cancer patients at baseline harbor tumors that are either devoid of immune cell infiltration or that are predominantly infiltrated by suppressive regulatory cell subtypes, including regulatory T cells (Tregs), regulatory B cells (Bregs), and myeloid-derived suppressor cells (MDSCs) [2,3]. These tumors are known as immunologically “cold”. Meanwhile, tumors infiltrated by tumoricidal effector immune cells, such as cytotoxic CD8 T cells and natural killer (NK) cells, are called immunologically “hot”. These tumor-infiltrating lymphocytes (TILs) correlate positively with augmented responses to immunotherapy and improved survival [4,5,6,7,8]. An immunologically inflamed (“hot”) TME exhibits robust antigen presentation and T cell activation, contributing to the expansion of tumor-specific CD8 TILs and the development of long-term antitumor memory responses [8,9]. In “cold” TMEs, cancer growth is immunologically unchecked, and thus, the recruitment of inflammatory immune cells into such tumors and their subsequent reactivation are imperative for tumor control [10,11]. Another, more recent phenotype, “immune-excluded or altered,” describes tumors in which CD8 T cells are confined to the periphery, impeding their infiltration into the tumor. The characteristics of “cold” and “altered” tumors imply a deficiency in the intrinsic antitumor capabilities, resulting in limited responses to immunotherapies such as ICIs [12].

Over the past eight years, the development of ICIs targeting PD-1/PD-L1 and CTLA-4 checkpoint axes has emerged as an unprecedented breakthrough for cancer treatment, promoting long-term tumor regression [13,14,15]. However, responses to such therapies have been demonstrated to be effective only in select patients, particularly those who harbor a “hot” TME at baseline [16]. Therefore, to increase responsiveness to ICI therapy, innovative solutions are needed to convert “cold” tumor microenvironments to “hot” ones. One proposed method is to introduce a natural enhancer and recruiter of TILs that would boost effector function and overall survival of effector cells in a “cold” TME, ultimately lowering the cancer-immune set point. For example, interleukin-15 (IL-15) is a stimulatory cytokine that drives effector activity, increases the proliferation and migration of immune cells into tissues [17,18,19,20], and enhances the survival of CD8 T cells and NK cells. IL-15 is a common gamma-chain (γc) cytokine that binds to IL-2Rβ/γc/IL-15Rα and is presented in trans on the surface of APCs via IL-15Rα. Further, IL-15 induces cytotoxic functions of T cells, promotes optimal T cell memory responses, induces granzyme B and perforin expression, and enhances antibody-dependent cellular cytotoxicity (ADCC) and macrophage activation [21]. Unlike IL-2, IL-15 neither maintains Tregs nor causes activation-induced cell death (AICD), thus reducing the stimulation of suppressive cells, including T regs and B regs [22,23,24,25].

Currently, several IL-15-delivering platforms are in preclinical and clinical development for cancer treatment—each with variations in IL-15 conformation that confer increased stability, bioavailability, and in vivo half-life [19]. An IL-15 complex of particular interest is a chimeric IL-15 receptor alpha (IL-15Rα) fused to an antibody Fc domain; when bound to the IL-15 cytokine (IL-15/IL15Rα-Fc), the IL-15 complex increases agonist activity and durability [26]. Combinatorial treatment with anti-PD-1 and IL-15 has increased proliferation, cytokine secretion, and cytotoxicity in CD8+ T cells and NK cells [27]. Although combination therapy with anti-PD-1 and IL-15 is generating efficacy, issues concerning tolerability with increasing dosage pose a high risk for immune-related adverse events.

Preclinical studies indicate that IL-15 superagonists have an advantage over IL-15 alone in mediating immune effects, specifically the expansion of NK and CD8+ T cells, without inducing significant toxicity [28]. For example, a fusion protein that combines IL-15 superagonist with anti-CD20 binding domains of rituximab (2B8T2) significantly reduced leukemic burden compared to rituximab therapy alone [29]. In addition, fusion of IL-15 superagonist to anti-PD-L1 binding domains to produce a bifunctional superagonist was administered subcutaneously in a mouse model of metastatic triple-negative breast cancer and colorectal cancer. It created a synergistic effect capable of reducing immune suppression while increasing the migration of peripheral NK and CD8+ T cells into the TME [21]. The fusion of an IL-15 superagonist with an anti-PD-L1 inhibitor elucidated a novel delivery mechanism for IL-15 via PD-L1 blockade. However, even with this potent synergy, antitumor immunity was short-lived in these experiments [30].

Our study uses a murine model of luminal B breast cancer (EO771) that lacks two significant biomarkers (Luminal B: ERα^−^, ERβ^+^, PR^+/−^, HER2^+^) [31]. Luminal B cancer is a commonly encountered subtype in breast cancer patients with poor prognoses [28,32]. Our study focuses on the effects of intraperitoneal administration of an anti-PD-1 mAb in combination with intratumorally delivered IL-15 complexes in luminal B tumor-bearing mice. Using Nanostring digital spatial profiling (DSP), it has been shown that luminal B breast cancers are enriched for immune marker expression related to T cells and immune activation compared with luminal ABC tumors, suggesting this subtype may respond favorably to our treatment regimen [33].

With this treatment regimen, we found that the combination induced long-lasting tumor immunity, with at least 85% of the mice remaining tumor-free for at least 50 days post-tumor implantation. To understand what happened within the TME during tumor regression, we examined the TME at the time tumors began to regress, compared with controls. We found that generating cytotoxic CD8+ TILs and NK cells capable of clearing tumors required two intratumoral doses of IL-15 complexes in combination with anti-PD-1 therapy. Additionally, this combinatorial regimen of two doses of IL-15 complex delivered intratumorally, in combination with a conventional anti-PD-1 treatment schedule, correlated with an increase in cytotoxic CD8+ T cells infiltrating the TME. Increased expression of genes involved in cytokine production, chemokine signaling, metabolism, TCR signaling, and co-stimulatory signaling was among the key pathways affected by this treatment regimen.

Furthermore, fluorescent and light-sheet microscopy show that IL-15 complexes and anti-PD-1 mAb are present in the tumor-draining lymph node. The ability of IL-15 complexes to migrate to the tumor-draining lymph node is critical for activating NK and CD8+ T cells. This activation leads to proliferation, the gain of effector function, and the ability of NK and CD8+ T cells to migrate into the TME, where they can release cytotoxic granules, resulting in tumor clearance [34]. Overall, using an orthotopic murine luminal B breast cancer model, our study identifies the immune signature of regressing tumors and the localization and in situ distribution of IL-15 complexes and anti-PD-1 mAb within immune cells in regressing and non-regressing luminal B tumors.

## 2. Results

### 2.1. A Single Dose of IL-15 Complex Fails to Enhance the Efficacy of Anti-PD-1 Therapy

**Figure 1:**
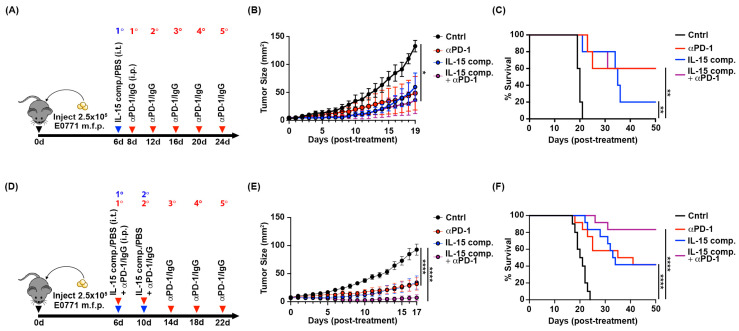
Intratumoral treatment with IL-15 complexes delays tumor progression, and two doses of IL-15 complex potentiate the efficacy of anti-PD-1 therapy in murine EO771 breast cancer. (**A**,**D**) Schematic diagrams indicating the frequency and timing of IL-15 complex and anti-PD-1 mAb administration. (**B**,**E**) Comparison of tumor sizes between treatment groups as measured by tumor area (L × W). Independent *t*-test and Tukey’s multiple comparison tests, * *p* ≤ 0.05, **** *p* < 0.0001. (**C**,**F**) Percent tumor-free survival. Log-rank test, ** *p* ≤ 0.005, **** *p* < 0.0001. Data represent one of three trials that used five mice per treatment group.

To determine if the IL-15 complex delivered intratumorally could improve anti-PD-1 therapy, C57BL/6 mice were first inoculated with the luminal B breast cancer cell line EO771 in the mammary fat pad. Figure 1A (Supplemental Figure S1A) is a schematic illustrating the treatment schedule given. Six days following tumor inoculation, when tumors were 5 mm^2^, mice were treated intratumorally (i.t.) with either PBS (vehicle control) (Supplemental Figure S1B) or IL-15 complexes (Supplemental Figure S1C). Two days following i.t. treatment, mice were treated intraperitoneally (i.p.) with anti-PD-1 mAb alone (Supplemental Figure S1D). A group of mice received a combination of anti-PD-1 mAb (i.p.) and IL-15 complex (i.t.) (Supplemental Figure S1E). Those animals that received anti-PD-1 mAb did so at four-day intervals for a total of five treatments (Figure 1A). Mice treated with either IL-15 complexes (i.t.) or anti-PD-1 mAb (i.p.) alone or an initial combination of the two showed significantly decreased tumor growth 19 days post-treatment as compared to control animals (Figure 1B). None of the treatments showed a significant difference in tumor growth (Figure 1B). A single dose of IL-15 complex intratumorally dramatically slowed the growth rate of 3/5 animals, with one animal rejecting the tumor long-term (greater than 50 days) (Supplemental Figure S1C). While animals treated with five doses of anti-PD-1 significantly slowed tumor growth; only three of five cleared the tumor (Supplemental Figure S1D). Although both single-modality treatments reduced tumor growth, combining a single dose of IL-15 complex with anti-PD-1 therapy did not show an additive effect on tumor growth or survival (Figure 1B and Supplemental Figure S1E). For example, the overall survival of mice treated with IL-15 complexes and anti-PD-1 mAb was identical to that of tumor-bearing mice treated with PD-1 blockade alone (Figure 1C).

### 2.2. Two Doses of IL-15 Complex Intratumorally Augment Anti-PD-1 Therapy in Murine EO771 Breast Cancer

To determine if a second dose of IL-15 complex could further enhance the efficacy of anti-PD-1 therapy, we next included a second intratumoral injection of IL-15 complex ten days following tumor inoculation (i.e., four days after the first dose of IL-15 complex) (Figure 1D). The treatment schedule we used to measure the effect of two doses of IL-15 complexes, either alone or in combination with anti-PD-1 mAb against EO771, is shown in Figure 1D. No animals cleared tumors in the control group treated with anti-IgG in PBS (Figure 1E,F and Supplemental Figure S1G). Intratumoral treatment with two doses of IL-15 complexes alone generated partial responses in 42% of mice (five out of twelve), in which these mice showed significantly delayed tumor progression compared to vehicle control (Figure 1E), while promoting tumor clearance in 58% of mice (seven out of twelve) (Figure 1F and Supplemental Figure S1H). Anti-PD-1 therapy alone significantly delayed tumor progression compared to control (Figure 1E), leading to tumor clearance in 58% of mice (seven out of twelve) (Figure 1F and Supplemental Figure S1I). Mice treated with the combination of anti-PD-1mAb (i.p.), and two doses of IL-15 complexes (i.t.) showed reduced tumor growth compared to tumors of mice in both single modality treatments and the control (PBS/IgG isotype) group (Figure 1E and Supplemental Figure S1J). However, no significant differences between treatments were observed at 17 days post-treatment.

At 50 days following initial treatment, overall survival was significantly extended in mice that received IL-15 complex alone, anti-PD-1mAb alone, or the combined treatment of IL-15 complex plus anti-PD-1 compared to vehicle control mice (0/12 mice tumor-free) (Figure 1F). Moreover, combined treatment of intratumoral IL-15 complexes with anti-PD-1mAb led to tumor regression and complete clearance of the EO771 tumor in 83% of mice (ten out of twelve) (Figure 1F and Supplemental Figure S1J), which is above the average overall response rate observed in non-breast solid tumors of patients treated with anti-PD-1mAb alone (~20%) [15].

### 2.3. Intratumoral Treatment with IL-15 Complexes Enhances Cytotoxic Immune Infiltrates in Anti-PD-1 Treatment Within EO771 Tumors, a Murine Luminal B Breast Cancer Model

**Figure 2:**
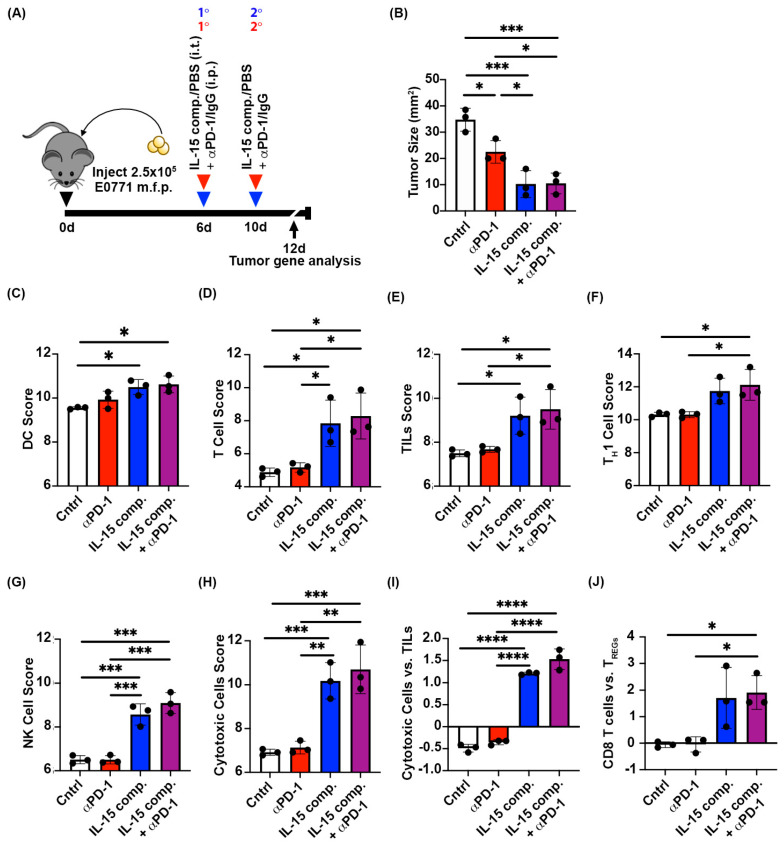
Intratumoral treatment with IL-15 complexes enhances DC and cytotoxic immune cells within murine EO771 breast cancer. (**A**) Schematic diagram of treatment scheduled prior to tumor gene analysis via Nanostring nCounter (multiplex panels from whole tumor lysates). (**B**) Tumor area measurements were taken at the time all tumors were collected for gene expression analysis. The raw score for dendritic cell (DC) gene expression (**C**), T cell gene expression (**D**), tumor-infiltrating lymphocyte (TIL) gene expression (**E**), T_H_1 cell gene expression (**F**), NK cell gene expression (**G**), and cytotoxic cell gene expression (**H**) are derived from the Nanostring^®^ PanCancer IO 360^TM^ Panel. The relative expression score of cytotoxic cells versus TILs genes (**I**) and CD8 T cells versus T_REG_ cell genes (**J**) (PanCancer Panel). One-way ANOVA and Tukey’s multiple comparison test, * *p* ≤ 0.05, ** *p* ≤ 0.005, *** *p* ≤ 0.0005, **** *p* < 0.0001. The data represent one trial that used three mice per treatment group.

To characterize the antitumor immune response in murine EO771 breast cancer induced by the two doses of intratumoral IL-15 complexes, we collected tumors at day twelve across treatment groups (two days following the second dose in groups receiving IL-15 complex treatment). RNA from whole tumors was analyzed using Nanostring^®^ PanCancer IO 360^TM^ panel (Figure 2A). At the time of tumor gene analysis, the average tumor sizes of mice treated with anti-PD-1 were significantly smaller than those of control mice (Figure 2B). In contrast, tumors in IL-15 complex-treated mice were significantly smaller than those in anti-PD-1 mAb- and control (PBS/IgG isotype)- treated mice (Figure 2B). Our data show that while anti-PD-1 treatment did not affect the abundance of dendritic cell (DC)-associated genes within the TME, IL-15 complexes delivered intratumorally significantly increased the abundance of DC-associated genes within the TME compared to control (PBS/IgG isotype) tumors (Figure 2C). This is relevant to generating a durable antitumor response as DCs cross-prime CD8 T cells, which is critical for developing effective antitumor immunity [35]. Furthermore, IL-15 complexes significantly increased T cell-associated gene scores compared with anti-PD-1 mAb alone or control (PBS/IgG isotype)- treated tumors (Figure 2D). High numbers of TILs have been used as a prognostic indicator to gauge patient responsiveness to immunotherapies and conventional chemotherapies [36]. Our findings show that intratumoral treatment with IL-15 complexes alone or combined with anti-PD-1 therapy significantly increases the abundance of TIL-associated genes compared to anti-PD-1 monotherapy and control (PBS/IgG isotype) treated tumors (Figure 2E). Similarly, positive outcomes in patients with advanced-stage tumors correlate with more significant lymphocyte tumor infiltration, particularly effector T cells [37].

Antitumor immune responses require CD4+ T_H_1 cells to mediate the cytotoxicity of CD8+ T effector cells and to generate tumor-specific T cell memory. Intratumoral treatment of luminal B tumors with IL-15 complexes combined with anti-PD-1 therapy significantly increases the expression level of T_H_1 cell-related genes (Figure 2F). A similar trend was observe with IL-15 complex treatment alone compared with anti-PD-1 therapy and control (PBS/IgG isotype), but did not reach statistical significance. Like CD8+ T cells, NK cells respond to IL-15 complexes, thereby enhancing cytotoxicity and effector activity. Intratumoral treatment with IL-15 complexes significantly increased the expression level of NK cell-related genes in the TME compared to anti-PD-1 monotherapy and control (PBS/IgG isotype) treatment (Figure 2G). In addition, intratumoral treatment with IL-15 complexes significantly increases the expression level of genes associated with cytotoxic cells compared to anti-PD-1 mAb and control (PBS/IgG) treatments (Figure 2H). Furthermore, this consistent cytotoxic response to intratumoral treatment with IL-15 complexes was maintained, with increased expression of TIL-related genes relative to tumors from mice treated with anti-PD-1 therapy alone or control (PBS/IgG isotype) mice (Figure 2I). Another critical indicator of patient responsiveness to immunotherapy is the relative abundance of CD8+ T cells to CD4+ T_REG_ cells within the TME. A high ratio of CD8+ T effector cells to CD4+ T suppressor cells indicates a greater proportion of CD8+ T cells to CD4+ T_REG_ cells and, thus, a higher likelihood of a more effective response to immune-enhancing therapies [38]. Here, we show that intratumoral treatment with IL-15 complexes, when combined with anti-PD-1 therapy, significantly enhances the expression level of CD8+ T cell-related genes relative to those of CD4+ T_REG_ cells in the TME compared to the CD8+ T cell: CD4+ T_REG_ ratio in tumor-bearing mice treated with anti-PD-1 therapy and control (PBS/IgG isotype) treatment (Figure 2J). A similar trend was observed in IL-15 complex treatment compared to anti-PD-1 therapy and control (PBS/IgG isotype) treatment but did not reach statistical significance. We found that specific genes associated with DCs, T cells, TIL, and NK cells were significantly upregulated in the TME following intratumoral treatment with IL-15 complexes and the combination treatment with PD-1 blockade.

### 2.4. Intratumoral Administration of IL-15 Complexes in Combination with Anti-PD-1mAb Therapy Enhances CD8+ T Cell and NK Cell Infiltration and Granzyme Genes Expressed in CD8+ TILs of Murine EO771 Breast Cancer

**Figure 3:**
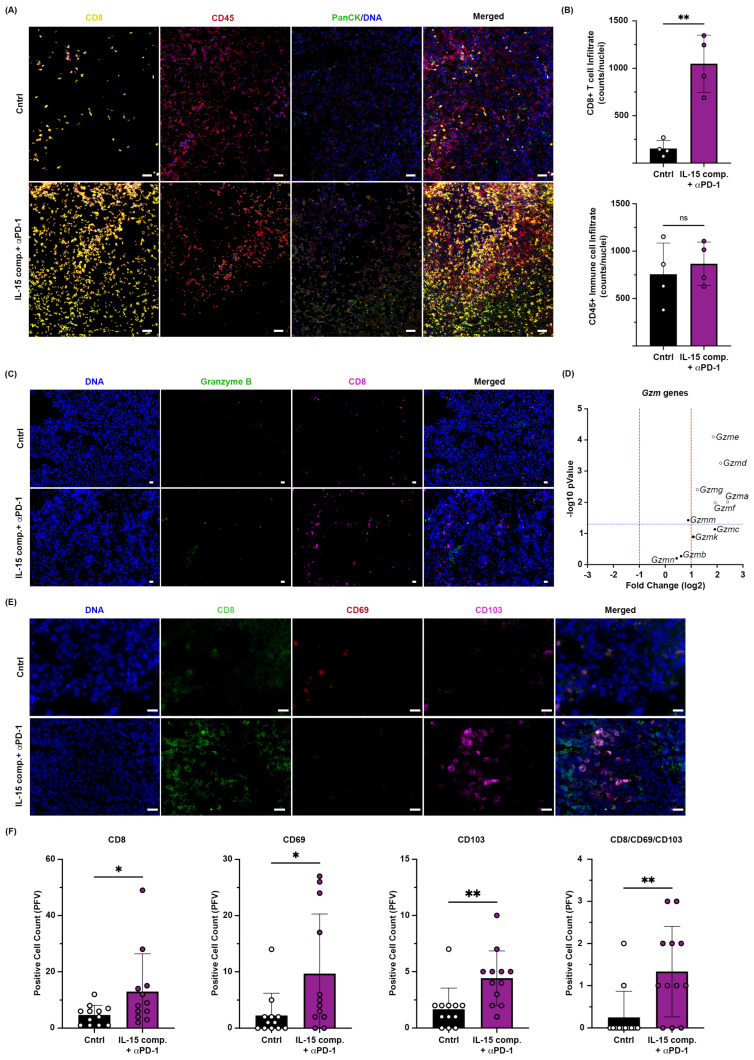
Nanostring GeoMx spatial protein profiling and Immunofluorescence staining. (**A**) Image of a tumor section from a representative EO771 tumor-bearing mouse that was either treated with PBS/IgG isotype or IL-15 complexes and anti-PD-1 and stained with CD8 (yellow), CD45 (red), pan-cytokeratin (green), and DNA (Blue); scale is 20 µm. (**B**) The quantification of CD8 and CD45 positive (+) cells from 5 ROIs. (**C**) Tumor OCT sections were analyzed 48 h after the second treatment with IL-15 complexes and anti-PD-1 mAb, stained for Granzyme B (green), CD8^+^ (magenta), and nuclei stained with Hoechst (blue); Scale is 20 µm. (**D**) Volcano plot with the Granzyme B genes that were upregulated in the TME of mice treated with IL-15 complexes and anti-PD-1 compared to the control (PBS/IgG isotype) group; red lines denote fold change greater than one, and blue lines denote statistical significance cutoff. Open circles distinguish genes with significant differential expression. (**E**) Conventional fluorescent microscopy of tumors injected with PBS (i.t.) and IgG isotype mAb (i.p.; (**top**) IL-15 complexes (i.t.) and anti-PD-1 mb (i.p.) dual treatment (**bottom**), stained for CD8^+^ (green), CD69^+^ (red), CD103^+^ (magenta), and nuclei stained with Hoechst (blue); Scale is 20 µm. (**F**) Cell count (per field of view) comparison between treated and PBS group for CD8^+^, CD103^+^, CD69^+^, and triple positive CD69^+^/CD103^+^/CD8. An independent *t*-test was performed; ns = not significant; * *p* ≤ 0.05; ** *p* ≤ 0.005. (**A**,**B**,**D**) Data represent one experiment that used four mice per treatment group, analyzed via GeoMx Digital Spatial Profiler (DSP). Each data point represents an individual tumor sample. (**C**,**E**,**F**) Data represent one of four trials, with 5–12 mice per treatment group, analyzed using BZ-X800 cell counter. An independent *t*-test was performed, * *p* < 0.05, ** *p* < 0.01. Each data point represents an individual tumor sample.

Next, we used fresh-frozen OCT-embedded tissue sections to characterize in situ how this combination treatment (IL-15 complexes + PD-1 blockade) can modulate effector responses within the TME. We relied on Nanostring^®^ GeoMx^®^ digital spatial profiling (DSP) and immunofluorescence. Serial sections from OCT blocks were obtained from EO771 tumor-bearing mice 48 h after the second treatment with either the IL-15 complex and anti- or control IgG antibody and stained for CD8, CD45, and PanCK to identify CD8+ TILs and tumor cells. Using the combination treatment of IL-15 complexes and anti-PD-1, we show increased CD8+ TILs (Figure 3A,B). Combining intratumor IL-15 complexes with systemic PD-1 blockade also increased granzyme B protein expression (Figure 3C). Although multiple granzyme genes in CD8+ TIL were increased with IL-15 complexes and anti-PD-1 treatment and correlated with the overall influx of CD8+ T cells (Figure 3B, C) within the tumor parenchyma, the gene expression of granzyme B was not increased as determined using Nanostring^®^ (Figure 3D). Granzymes, including Granzyme B, are known to be subject to post-transcriptional regulation, meaning their protein levels can vary independently of their gene expression levels. One possibility for the discrepancy between the Nanostring and our Immunofluorescence data is that although the RNA transcripts of multiple granzymes might be upregulated, Granzyme B might be specifically processed or modified (e.g., glycosylated or cleaved) for its function, making it more detectable in immunofluorescence.

We next used fluorescent staining to confirm the Nanostring^®^ data. Tumors were obtained 48 h after the second treatment of IL-15 complexes (i.t.) and anti-PD-1mAb (i.p.), and tissue was obtained 48 h after the second treatment of IL-15 complexes (i.t.) and anti-PD-1mAb (i.p.) and placed in OCT, sections were cut and stained for CD8+ T lymphocytes, CD69, CD103, and Granzyme B. Using fluorescent staining we found an increased expression of CD8^+^, CD69^+^, and CD103^+^ in IL-15 complexes and anti-PD-1 mAb co-treated group as compared to the control (PBS/IgG isotype) group (Figure 3E). The initial measurement used individual cell counts obtained with a Keyence BZ-800 analyzer and was standardized to reflect the number of nuclei present (Figure 3F). Consequently, this combination treatment enhanced infiltration of CD8+ T cells and NK cells into the tumor and increased granzyme expression.

### 2.5. Intratumoral Treatment with IL-15 Complexes Modulates Immune Activation Signaling and Metabolic Pathways Within Murine EO771 Breast Cancer

**Figure 4:**
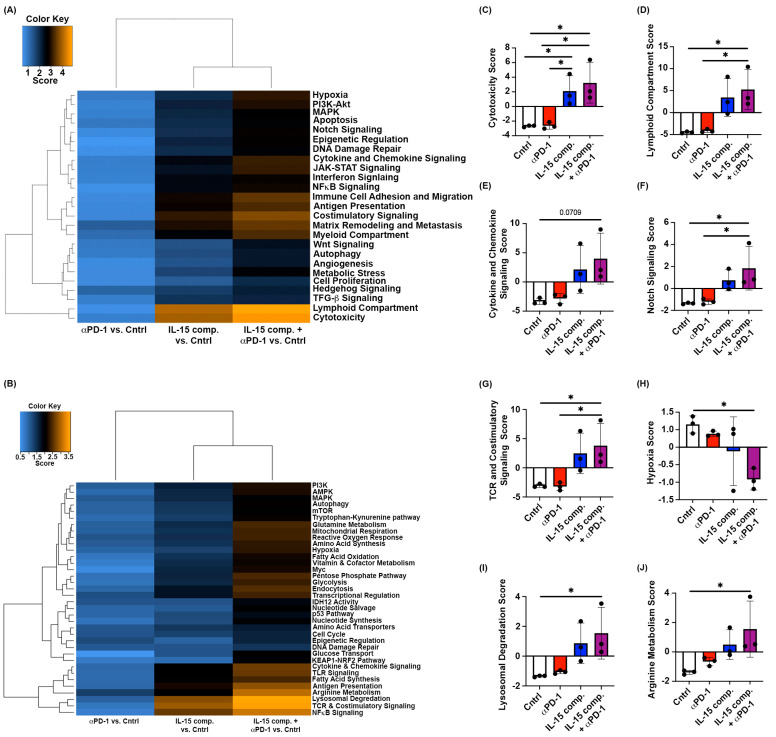
Tumor gene analysis of the combinatorial treatment with IL-15 complexes and anti-PD-1 therapy using Nanostring nCounter^®^ multiplex panels from whole tumor lysates. (**A**) Heat map for the PanCancer IO 360^TM^ panel. (**B**) Heat map for the metabolism panel. (**C**) Raw cytotoxicity-associated gene expression score (PanCancer Panel). (**D**) Raw Lymphoid compartment gene expression score (PanCancer Panel). (**E**) Raw cytokine and chemokine signaling gene expression score (PanCancer Panel). (**F**) Raw Notch signaling-associated gene expression score (PanCancer Panel). (**G**) Raw TCR and co-stimulatory signaling gene expression score (Metabolic Pathways panel). (**H**) Raw hypoxia-associated gene expression score (Metabolism Panel). (**I**) Raw lysosomal degradation-associated gene expression score (Metabolism Pathway panel). (**J**) Raw arginine metabolism gene expression score (Metabolism Panel). Data are from one experiment with three mice per group. An independent *t*-test was performed, * *p* < 0.05.

Given the upregulation of genes associated with enhanced pro-inflammatory cells and cytotoxicity, we next sought to measure changes in major pathways involved in immune activity and metabolism. For this, we utilized EO771 tumors from the m.f.p. of mice two days following the second dose of IL-15 complexes and/or anti-PD-1 treatment. RNA from the whole tumor was analyzed using Nanostring^®^ PanCancer IO 360^TM^ panel (Figure 2A). At the time of tumor gene analysis, the average tumor sizes of mice treated with control, anti-PD-1 mAb alone, IL-15 complexes alone, or a combination of anti-PD-1 mAb and IL-15 complexes are shown (Figure 2B). Figure 4A,B represents heat maps generated using the PanCancer IO 360^TM^ or the metabolism Nanostring^®^ panels, respectively. An antitumor immune response capable of clearing tumors and distant metastasis necessitates early induction of lymphoid cells and effective T-cell signaling. Similarly to the cytotoxic cell scores (Figure 2H,I), intratumoral treatment with IL-15 complexes significantly upregulated genes associated with cytotoxicity within the TME compared to anti-PD-1 therapy alone and control (PBS/IgG isotype) treatment (Figure 2C and Figure 4C).

We show that intratumoral treatment with IL-15 complexes combined with anti-PD-1 therapy significantly increased the expression level of genes related to the lymphoid compartment in the TME compared to anti-PD-1 monotherapy and control (PBS/IgG isotype) treatment (Figure 4D). A similar trend was observed with IL-15 complex treatment alone compared with anti-PD-1 therapy and control (PBS/IgG isotype) but did not reach statistical significance. A significant barrier to tumor infiltration by pro-inflammatory and important tumoricidal lymphocytes is the absence of chemotactic and immunogenic signals in the TME and draining lymph nodes. Although intratumoral treatment with IL-15 complexes did not induce significant upregulation in the cytokine and chemokine signaling pathway score, it did show an increasing trend in these treatment groups compared with groups treated with anti-PD-1 monotherapy and control (PBS/IgG isotype) (Figure 4E and Supplemental Figure S2B). In line with DC-associated genes being upregulated (Figure 2C), we also found genes associated with Notch signaling and TCR and co-stimulatory signaling to be upregulated in the double treatment group (Figure 4F and Figure 4G and Supplementary Figures S2A and S2G, respectively), which is required for activation and effector function of CD4^+^ and CD8^+^ T cells.

Our data show, for the first time, that intratumoral treatment with IL-15 complexes combined with anti-PD-1 therapy downregulates hypoxia-related genes compared with control (PBS/IgG isotype) treatment (Figure 4H and Supplementary Figure S2D). Studies have elucidated that lysosomes can enable cancer cells to cope with environmental stress and aid in cancer development [39,40]. Our data reveal that combining anti-PD-1 with intratumoral IL-15 complexes significantly upregulates genes associated with lysosomal degradation compared to control (PBS/IgG isotype) treatment (Figure 4I and Supplementary Figure S2F).

Interestingly, intratumoral treatment with IL-15 complexes combined with anti-PD-1 therapy significantly upregulates genes associated with arginine metabolism compared with control (PBS/IgG isotype) treatment (Figure 4J and Supplementary Figure S2E). It has been shown that arginine metabolism is critical for T cell activation, survival, and driving antitumor activity [41]. Thus, specific genes associated with TCR and co-stimulatory signaling were significantly upregulated in the TME following intratumoral treatment with IL-15 complexes. These included *gzmd*, *zap70*, *cd3d*, *cd27*, and *cd28.* Intratumoral treatment with IL-15 complexes also induced differences in the expression of specific cytokine and cytokine signaling genes when combined with anti-PD-1mAb. Overall, the heat maps in Supplementary Figure S2 for treatments with IL-15 complexes or IL-15 complexes plus anti-PD-1 therapy show a significant fold change in gene expression compared to control. While many of these gene fold changes show similarities between the two groups treated with IL-15 complexes, several genes per gene set show significant fold changes following combination treatment with IL-15 complexes and PD-1 blockade compared to IL-15 complex monotherapy.

### 2.6. Increased Gene Expression of Multiple Tumor-Homing Receptors in CD8+ TILs Following Combinatorial Treatment with IL-15 Complexes and PD-1 Blockade

**Figure 5:**
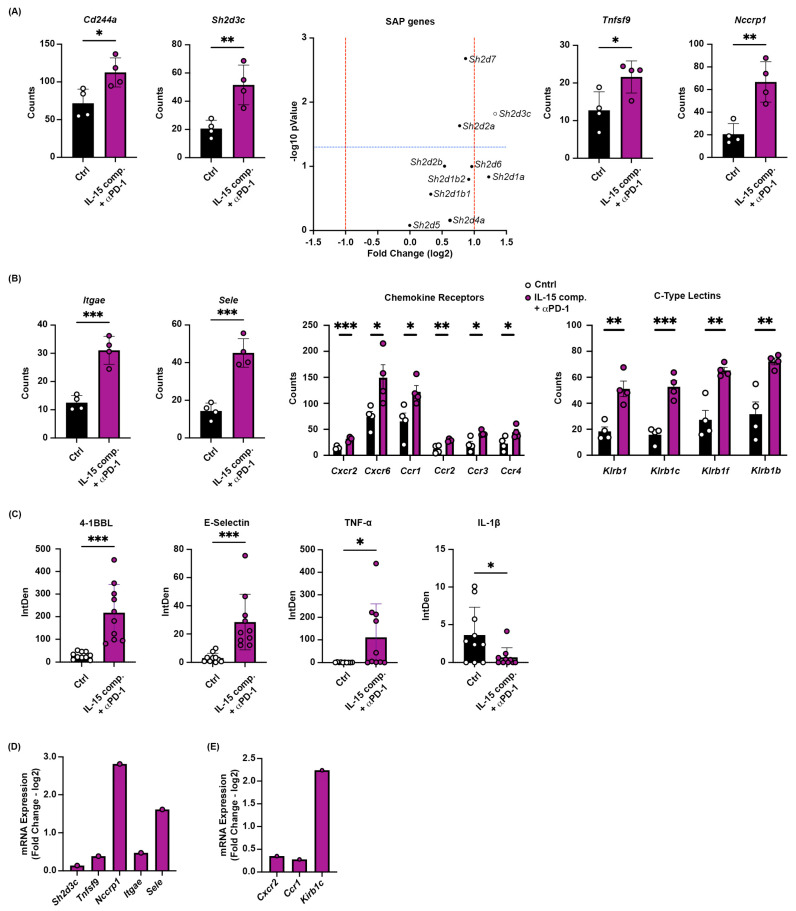
Differential expression of genes involved in immune activation, migration, and tissue residency of CD8 TILs. The treatment schedule prior to tumor gene expression analysis via Nanostring GeoMx DSP genomics using the Mouse Whole Transcriptome Atlas was the same as in Figure 2A. RNA expression data is specifically measured from identified CD8 T cells from tissue sections. (**A**) Cd244a, SAP, and co-stimulatory protein expression on CD8 T cells as determined by RNA Nanostring analysis; red lines denote fold change greater than one and blue lines denote statistical significance cutoff. Open circles distinguish genes with significant differential expression. (**B**) Integrins Alpha-E (Itage) and E-selectin (Sele) expression. These data are from one experiment with four mice per group. Each data point represents an individual tumor sample. (**C**) 4-1BBL, E Selectin, TNF-α, and IL-1β expression. The data represent one of three trials, with four to twelve mice per treatment group. An independent *t*-test was performed; * *p* ≤ 0.05, ** *p* ≤ 0.005, *** *p* ≤ 0.0005. (**D**,**E**) mRNA expression is measured from tumor RNA isolates of IL-15 complex and anti-PD-1 mAb-treated mice and PBS/IgG isotype–treated mice. The 2(−Delta Delta C(T)) method of analysis was performed using real-time quantitative PCR data, and the log2 fold change plot represents the differential expression between control and treated groups from the above panels. The data represent 10 mice per group from an experiment independent of the Nanostring data.

**Figure 6:**
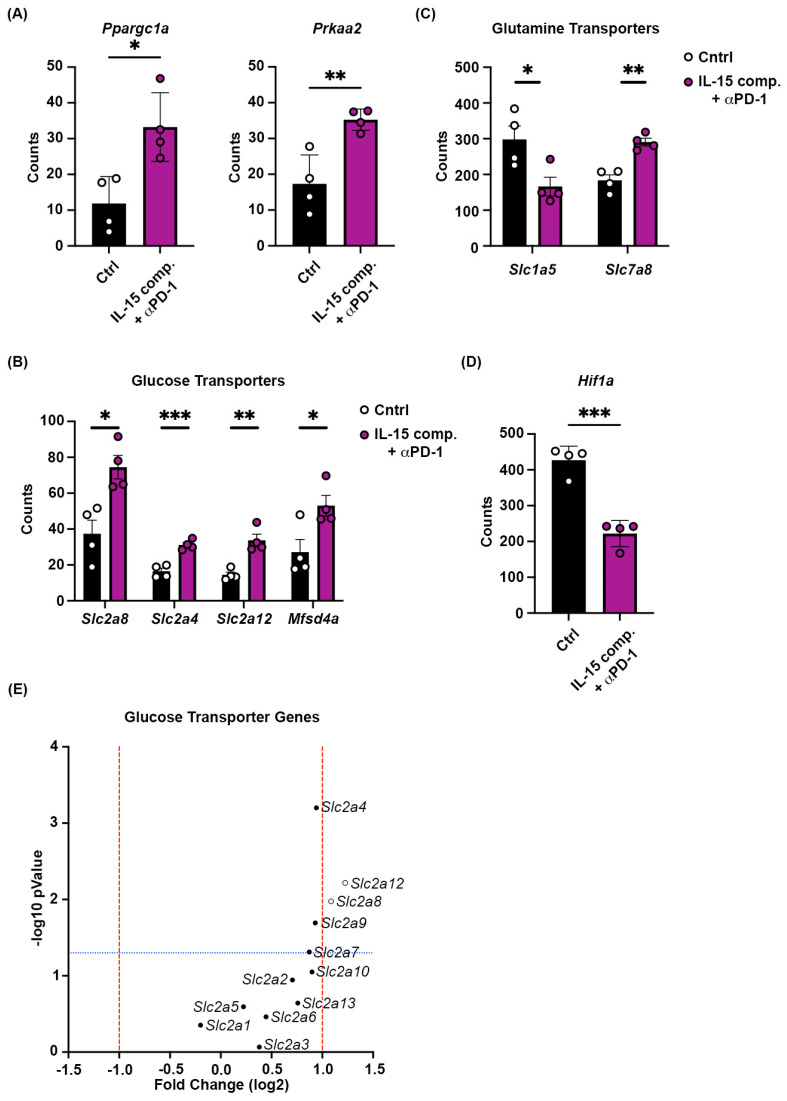
Combining intratumoral IL-15 complexes with PD-1 blockade upregulates gene expression of *ppargc1a* and glucose transport proteins in CD8^+^ T cells and reduces hypoxic gene expression. Treatment schedule prior to tumor gene expression analysis via Nanostring GeoMx^®^ DSP genomics analysis using Mouse Whole Transcriptome Atlas was the same as in Figure 2A. (**A**) Expression of mitochondrial biogenesis genes, (**B**) mRNA, (**C**) Glucose transporter, (**D**) glutamine transporters, and (**E**) Hypoxic marker Hif1a; red lines denote fold change greater than one and blue lines denote statistical significance cutoff. Open circles distinguish genes with significant differential expression. The data are from one experiment with four mice per group. Each data point represents an individual tumor sample. An independent *t*-test was performed, * *p* ≤ 0.05, ** *p* ≤ 0.005, *** *p* ≤ 0.0005. mRNA expression is measured from tumor RNA isolates of IL-15 complex and anti-PD-1 mAb- and PBS/IgG isotype–treated mice. The 2(−Delta Delta C(T)) method of analysis was performed using real-time quantitative PCR data, and the log2 fold change plot shows differential expression between the control and treated groups in panels (**A**) and (**C**–**E**).

Next, we wanted to investigate the genes involved with immune activation, migration, and tissue residency. We used RNA from whole-tumor lysates isolated from tumor-bearing (EO771) mice that had received IL-15 complexes and anti-PD-1 mAb or an IgG isotype control antibody for these experiments. Analysis was performed using RNA from whole tumors using the Nanostring^®^ PanCancer IO 360^TM^ panel, and mRNA expression was confirmed by quantitative reverse transcription polymerase chain reaction (RT-qPCR). CD8 TILs were identified using GeoMx DSP analysis of the whole mouse transcriptome and morphological immune fluorescence markers to identify CD8 T cells. For immune activation, we observed increased expression of Cd244a (2B4) in the combination therapy group (IL-15 complexes + anti-PD-1 mAb) compared with the control (PBS/IgG isotype) group (Figure 5A). Cd244a is known to play a role in NK activation, which would be indispensable for tumor immunity [42]. In addition, the level of Cd244a expression and its activation signaling depend on the level of SAPs (Figure 6A). Moreover, this combination therapy increased the expression of multiple tumor-homing receptors and the SLAM-associated protein (SAP) in CD8+ TILs, including *sele* (Selectin-E), Itgae (CD103), and SAP-related genes, which are essential for T cell migration signaling and antigen-driven T cell activity, respectively.

SAP (Sh2d1a) is an adaptor molecule important for T-cell signaling. It has recently been shown to interact with the signaling lymphocytic activation molecules (SLAM) receptors in the context of self-tolerance. SAP binds to the proximal tyrosine residue on CD28, thereby inhibiting PD-1 signaling [43]. Our data show that several SAP genes, including Sh2d3c, are significantly upregulated in the double-treatment group compared to the control group (Figure 5A). Sh2d3c acts as an adaptor protein that mediates cell signaling, regulates cell adhesion and migration, and modulates immune responses [44]. The tumor necrosis factor ligand superfamily member 9 (TNFSF9, 4-1BBL) is a co-stimulatory receptor on T lymphocytes and is overexpressed in the double-treatment group compared with controls (Figure 5A). The interaction of 4-1BBL expressed on APCs with its co-stimulatory receptor on CD4^+^ and CD8^+^ T cells induces antigen presentation and the generation of cytotoxic T cells [45]. The upregulation of Nccrp1 (Figure 5A) may also contribute to proliferation and possibly cytotoxicity, thereby influencing TIL function.

Our data show that the CD103 (Itage) and E-selectin (sele) were increased in CD8 TILs in those animals that received both IL-15 complexes and anti-PD-1 therapy (Figure 5B). These integrins regulate cellular growth, proliferation, migration, signaling, cytokine activation, and release. Furthermore, mounting evidence suggests that a subset of CD8+ TILs that express CD103 are tissue residents, and this population is associated with a more favorable survival outcome [46]. The combinatorial treatment increased the expression of chemokine and cytokine receptor genes, cxcr3 and cxcr6, which are essential for recruiting NK and CD8+ T cells into the TME (Figure 5B). In addition, C-type lectin-like receptors were also upregulated in treated animals compared to controls (Figure 5B).

To visualize the significant genes obtained through Nanostring^®^ analysis, we used tumors from mice 48 h after their second treatment regimen. (the same time-point that tumors were analyzed using Nanostring^®^. Tumors were harvested and placed in OCT, and serial sections were cut and stained. Fluorescence microscopy was used to analyze and determine the integrated fluorescence density for each biomarker (Figure 5C). The data show an increase in 4-1BBL (Tnfsf9), E-selectin (Sele), and TNF-α, as well as a reduction in IL-1β fluorescent density, in IL-15 complex and anti-PD-1 mAb-treated tumors versus PBS/IgG isotype control (Figure 5C). Eight significant genes were selected, and mRNA expression was confirmed by RT-qPCR in IL-15 complex- and anti-PD-1 mAb-treated tumors (Figure 5D,E). Thus, these data show that the combination treatment induced multiple tumor-homing receptors in CD8+ TILs, such as E-selectin, *Itage*, and SAP, which are important for T cell trafficking to tumors.

### 2.7. Combining Intratumoral IL-15 Complexes with PD-1 Blockade Regulates Gene Expression of Critical Mitochondrial Biogenesis Gene, Peroxisome Proliferator-Activated Receptor Gamma Coactivator Alpha (ppargc1a), and Glucose Transport Proteins in CD8+ T Cells to Reduce Hypoxia

Our results using RNA from whole tumors show that combining IL-15 complexes and anti-PD-1 mAb increases both ppargc1a and prkaa2 (Figure 6A), which coincides with the increase in the number of CD8+ TILs (Figure 4A,B). Ppargc1a and prkaa2 mRNA expression was also confirmed by RT-qPCR in IL-15 complex and anti-PD-1 mAb-treated tumors (Figure 6B). Our combination of IL-15 complexes and anti-PD-1 therapy significantly increased the expression of several glucose transport proteins in CD8^+^ T cells (Figure 6C). Furthermore, there appears to be a trend toward increased gene expression of Slc2 family members. While this therapy differentially modulates the gene expression of glutamine transport proteins on CD8 TILs (Figure 6D). We also identified HIF-1α to be downregulated with the combination treatment that enhances antitumor immunity (Figure 6E). HIF-1α is a hypoxic marker elevated in several cancers and is associated with poor prognosis and overall survival [47,48,49,50]. Thus, regulating the expression of PGC-1α, Slc2, and HIF-1α using IL-15 complexes and anti-PD-1 blockade could lead to increased functional CD8+ TILs and, ultimately, an increase in durable therapeutic responses.

### 2.8. Receptor and Ligand Expression on Tumors Treated with IL-15 Complexes and Anti-PD-1 mAb

**Figure 7:**
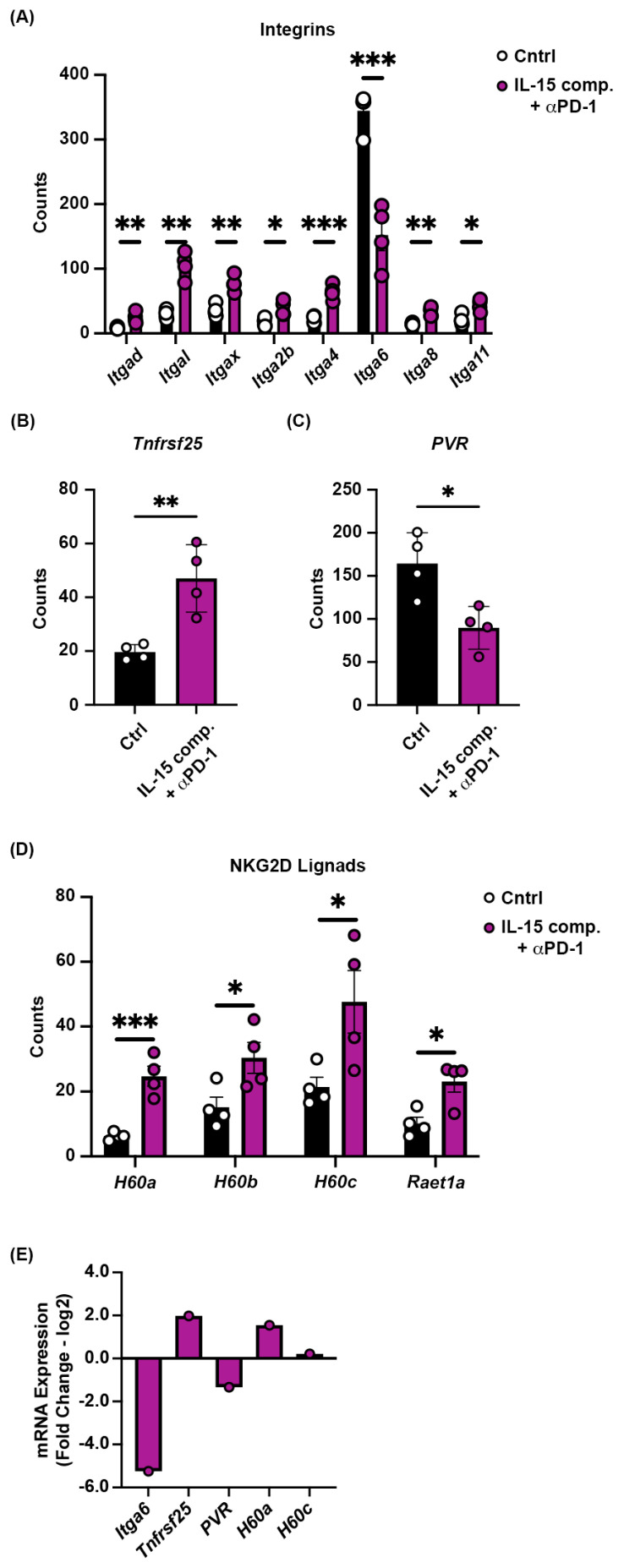
Integrins, DR3, PVR, and NKG2D receptor expression. Treatment schedule prior to tumor gene analysis via GeoMx^®^ DSP genomics analysis using Mouse Whole Transcriptome Atlas was the same as in Figure 2A. (**A**) Integrin expression, (**B**) DR3(Tnfrsf25) expression, (**C**) PVR expression, and (**D**) NKG2D receptor expression on the tumors of PBS/IgG isotype-treated and IL-15 and anti-PD-1 treated animals two days after their last treatment ~14 days post-inoculation. The data are from one experiment with four mice per group. Each data point represents an individual tumor sample. An independent *t*-test was performed, * *p* ≤ 0.05, ** *p* ≤ 0.005, *** *p* ≤ 0.0005. (**E**) mRNA expression is measured from tumor RNA isolates of IL-15 complex and anti-PD-1 mAb- treated mice and PBS/IgG isotype-treated mice. The 2(−Delta Delta C(T)) method of analysis was performed using real-time quantitative PCR data, and the log2 fold change plot represents the differential expression between control and treated groups from the above panels.

We next investigated the expression of integrins, ligands, and receptors that were differentially expressed on the tumor 48 h after the second treatment with either PBS/IgG isotype or IL-15 complexes, or after anti-PD-1 therapy. RNA from whole tumors was analyzed using Nanostring^®^ PanCancer IO 360^TM^ panel. For these experiments, the genes were specifically identified in tumor cells using GeoMx DSP analysis of the whole mouse transcriptome and morphological immune fluorescence to identify tumors via PanCK expression. We found an upregulation of integrins expressed in cancer (PanCK), including itgad, itgal, itgax, itga2b, itga4, itga8, and itga11 (Figure 7A). On the other hand, itga6 was significantly downregulated in tumors from animals that received both IL-15 complexes and anti-PD-1 mAb compared with controls (Figure 7A). Down-regulation of hypoxia-associated genes along with itga6 in response to combined treatment with IL-15 complexes and anti-PD-1 mAb therapy demonstrates how immune control of breast cancer by killing tumor cells and disrupting tumor metabolism can, in turn, attenuate tumor cell adhesion, stemness, metastasis, and even drug resistance via the downregulation of itga6 in tumor cells [51,52].

The death receptor 3 (DR3), also known as the tumor necrosis factor receptor superfamily member 25 (TNFRSF25), was upregulated in animals treated with IL-15 complexes and anti-PD-1 mAb (Figure 7B). DR3 mediates apoptotic signaling and differentiation. It has been reported to provide co-stimulatory signals for activated lymphocytes [53]. Poliovirus receptor (PVR, CD155), a member of the nectin-like family of proteins, is involved in critical cellular processes, such as adhesion, contact inhibition, migration, proliferation, and the immune response [54]. Our data show that combining IL-15 complexes with anti-PD-1 mAb reduces PVR expression (Figure 7C).

The natural killer group 2 member D (NKG2D) is an essential activating receptor with multiple ligands (NKG2DLs). The retinoic acid transcript-1/UL-16 binding proteins (RAET1/ULBP) 1–6 play a decisive role in antitumor immune surveillance [55]. In addition, NKG2D binds to retinoic acid early inducible 1 (Rae1) and H60 and ULBP-like transcript 1 (Mult-1). NKG2D-NKG2DL signals induce degranulation and/or cytokine production in cytotoxic effector cells [56,57]. We show that IL-15 complexes and anti-PD-1 therapy induce the expression of NKG2D ligand genes H60a, H60b, H60c, and Raet1a in murine luminal B breast cancer cells (Figure 7D). To confirm the Nanostring data, we chose those genes that were most significantly changed, including Itga6, Tnfrsf25, PVR, H60a, and H60c, and mRNA expression was confirmed by RT-qPCR in IL-15 complex and anti-PD-1 mAb treated tumors (Figure 7E).

### 2.9. The Spatial Distribution of IL-15 Complexes and Anti-PD-1 mAb Therapy in the TME and Tumor-Draining Lymph Nodes (TDLN)

**Figure 8:**
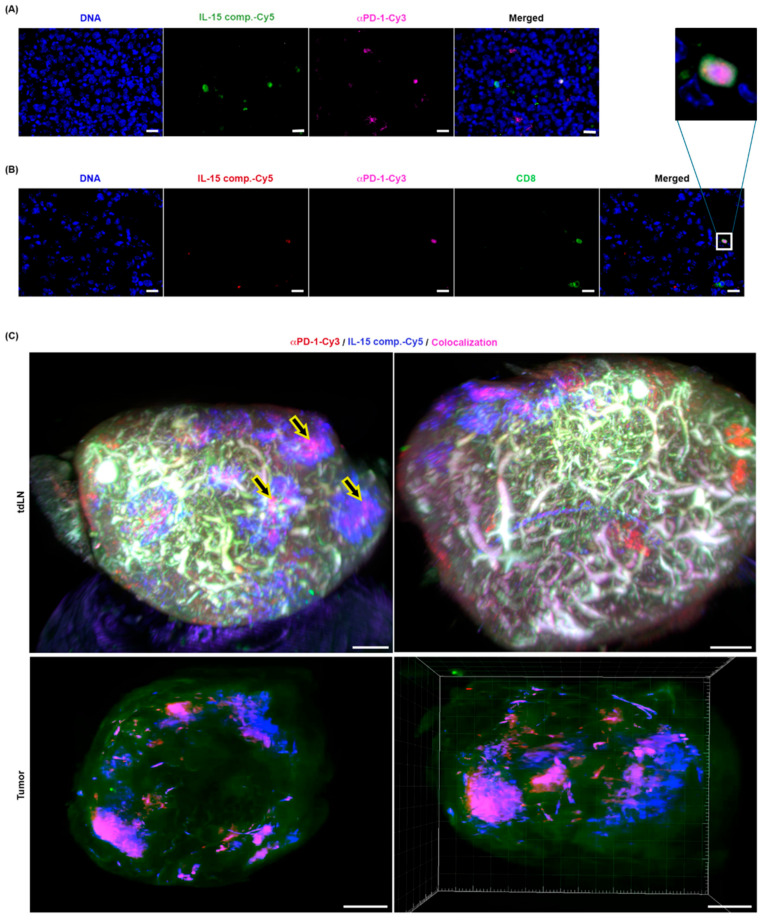
Colocalization of IL-15 complex-Cy5 and anti-PD-1-Cy3 in tumors and draining lymph nodes in murine E0771 breast cancer. (**A**) Colocalization of fluorescently labeled IL-15 complex-Cy5 (green) and anti-PD-1-Cy3 (magenta) in the tumor microenvironment. Nuclei stained with Hoechst (blue). Scale is 20 µm. (**B**) Colocalization of IL-15 complex-Cy5 (red), anti-PD-1-Cy3 (magenta), and CD8-488 (green) in the Tumor microenvironment. The inset shows a zoom in on a cell representing colocalization from the merge. Nuclei stained with Hoechst (blue). Scale is 20 µm. (**C**) Light Sheet Fluorescent Microscopy (LSFM) of representative whole-cleared draining lymph nodes (**top**) and whole-cleared tumors (**bottom**) intratumorally injected with IL-15 complex-Cy5 (blue) and intraperitoneally injected with anti-PD-1-cy3 (red), colocalization indicated in magenta (yellow arrows). 3D images processed using IMARIS. Scale is 200 µm.

To characterize the distribution of IL-15 complexes and anti-PD-1 mAb in situ and track the interactions with immune cells within the tumor, we fluorescently labeled IL-15Rα-Fc with Cy5 and anti-PD-1 mAb with Cy3. C57BL/6 mice were first inoculated with the EO771 luminal B breast cancer cell line in the mammary fat pad. When tumors reached ~5 mm^2^ in size (6 days post tumor inoculation), mice were treated with either control antibody (anti-IgG isotype in PBS (i.p.), IL-15 complexes (IL-15Rα-Fc labeled with Cy5) (i.t.), and anti-PD-1 mAb (labeled with Cy3) (i.p.) or a combination of anti-PD-1 mAb (i.p.) and IL-15 complexes (i.t.) or PBS/IgG isotype (i.t.). Mice were taken down 12 days after the initial inoculation of EO771 tumors or 2 days after the second IL-15 complex and anti-PD-1 treatment, and the tumor and tumor-draining lymph node were removed and placed in OCT blocks. Serial sections were cut, and fluorescently labeled IL-15 complex and anti-PD-1 mAb were imaged in the TME (Figure 8A). Colocalization of IL-15 and anti-PD-1 with CD8^+^ TILs was identified (Figure 8B insert). Within the draining tumor lymph node, anti-PD-1 mAb and IL-15 complexes colocalize (Figure 8C). Based on fluorescence staining, IL-15 intensity is localized to the cortical and paracortical regions of the lymph node. In addition, areas devoid of anti-PD-1 staining and IL-15 complexes are present. In the TME, IL-15 complexes and anti-PD-1 mAb localization differ from those in the TDLN, where IL-15 complexes appear to be maintained proximal to the injected area (tumor interior). At the same time, anti-PD-1 mAb staining is localized to the tumor surface (Figure 8C).

### 2.10. Complete Responses to Immunotherapy Generate Durable Immune Responses Against Murine Luminal Breast Cancer

**Figure 9:**
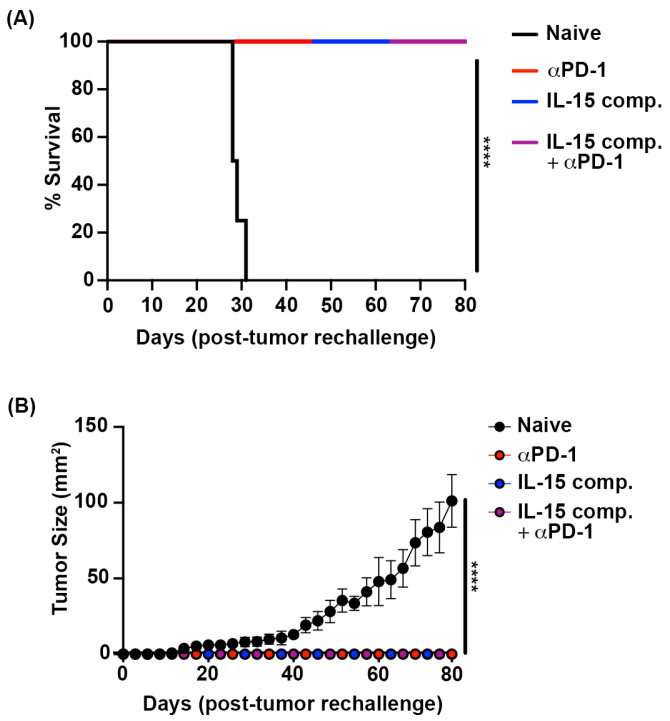
Treatment with IL-15 complexes and/or anti-PD-1 therapy protects against tumor rechallenge in the EO771 mouse breast cancer model. EO771 tumor-bearing mice that were treated with IL-15 complexes and/or anti-PD-1 therapy, cleared the tumor, and survived were rechallenged with 2.5 × 10^5^ EO771 tumor cells in the left m.f.p., at least 100 days following the first tumor injection on the right m.f.p. Naïve C57B6 mice previously unchallenged with tumor were used as controls to ensure proper tumor take. (**A**) Percent tumor-free survival. Log-rank test, **** *p* ≤ 0.0001. (**B**) Comparison of tumor progression between naïve challenged and rechallenged mice from treatment groups. Tumor sizes were compared when the first naïve mouse reached maximum tumor size (≥150 mm^2^) as measured by tumor area (L × W). An independent *t*-test was performed, **** *p* < 0.0001. The data represent five naïve C57B6 mice and all surviving mice (17) from two previous trials that were challenged with EO771 tumor cells.

To determine if EO771 tumor-bearing mice treated with IL-15 complexes and/or anti-PD-1 therapy effectively cleared tumors and were protected from further tumor regrowth, we rechallenged these animals. Mice were rechallenged with EO771 tumor cells injected on the left m.f.p., at least 100 days following the first tumor injection on the right m.f.p. Naïve C57B6 mice were challenged with EO771 tumor to control for proper tumor engraftment. Our data show that at least 80 days after rechallenging, 100% of mice survived (Figure 9A) and remained tumor-free (Figure 9B). Interestingly, all tumor-free mice on rechallenge were protected (Figure 9).

## 3. Discussion

Immune landscapes of breast cancers are heterogeneous and primarily dependent on cancer subtype, staging at the time of treatment, and initial line of therapy (e.g., radiation, chemotherapies, and surgery). Another factor that complicates treatment success is the differing responses to immunotherapies. Given the low efficacy of monotherapy and modest efficacy when combined with radiation/chemo, many clinical trials have tested combinatorial immunotherapies. Thus, there is a great need for predictive biomarkers of responsiveness to tailor treatments to target elements within the TME that can overcome immune deficits and expand clinical responses to immunotherapies in breast cancer patients. Numerous studies have evaluated the efficacy of monomeric IL-15 and IL-15 superagonists, alone or combined with PD-1/PD-L1 blockade, in mice and humans [1,58,59]. Previous studies have shown a much longer half-life and greater retention of the IL-15 superagonist in lymphoid tissues than monomeric IL-15, consistent with its effective immunostimulatory and antitumor activities in vivo [60]. Prior studies have delivered an IL-15 superagonist intravenously or subcutaneously and have used dosing regimens in combination with PD-1/PD-L1; however, these studies have shown that increased survival and tumor immunity were short-lived [30]. Another study developed a conditionally activated anti-PD-L1/IL-15 immunocytokine prodrug. This prodrug masks IL-15 with steric hindrance, providing a way to reduce systemic toxicities associated with the delivery of anti-PD-L1/IL-15. Furthermore, upon specific proteolytic cleavage within the TME, the prodrug releases an active IL-15 superagonist, inducing a potent antitumor response and converting cold tumors into hot tumors. Moreover, the prodrug synergized with oncolytic virus treatment in B16 melanoma [61]. We used the original IL-15 complexes that are more potent, but delivered them intratumorally to prevent the adverse side effects shown with intravenous administration. Subsequently, our study established that two intratumoral doses of IL-15 complexes in combination with the checkpoint inhibitor against PD-1 can convert the resistant EO771 luminal B TME into one more susceptible to complete immune control, as observed by a dramatic reduction in tumor size and metastatic centers, and 90% tumor clearance in mice without recurrence. Our results demonstrate that two doses of IL-15 complexes are required to induce this robust response, likely due to the IL-15 complexes’ pharmacokinetics and/or pharmacodynamics. For example, depending on IL-15’s half-life, complexes are administered in the TME dual treatment four days apart, combined with anti-PD1 therapy, which improves the therapeutic window. In addition, a second intratumoral delivery of IL-15 complexes may improve penetrance throughout the tumor, and it is possible that double treatment provides enough IL-15 complexes throughout the TME and extends time under treatment compared to a single dose; thus, enhancing the antitumor immune response and overall efficacy. Our findings are in line with more recent data that show that an anti-PD-1-fused IL-15 cytokine was more efficacious than an IL-15 superagonist either alone or in combination with anti-mPD-1 [62]. However, the difference between their study and ours is that they show antitumor response only up to 25 days in both a mouse model of melanoma (B16-F10) and a colon cancer (MC38) model. In contrast, we show using the luminal B mouse model (EO771) that tumor immunity is sustained for up to 50 days and that, on rechallenge, these mice are protected from further tumor insults.

Prior studies have shown that effective anti-PD-1/PD-L1 therapies primarily activate peripheral CD8 T cells that migrate to the TME to mediate killing, rather than reinvigorating CD8+ T cells within the tumor [63]. Thus, the most novel finding in our studies was that when IL-15 complexes were delivered via the intratumoral route, the cytokine reached the tumor-draining lymph nodes, activating CD8+ T cells and other immune cells and inducing their migration into the TME. Using conventional fluorescent and light-sheet fluorescent microscopy, we show that two doses of intratumoral IL-15 complexes coupled with anti-PD-1 mAb reach the tumor-draining lymph node, where they can engage CD8+ T cells and other immune cells, resulting in their activation. Moreover, we show that IL-15 complexes primarily localize to the paracortical (black arrows) and, to a lesser extent, the cortical regions (white arrows) of the tumor-draining lymph nodes, and anti-PD-1 mAb is detected in the tumor-draining lymph nodes, where they colocalize in primary and secondary follicles (gray arrows). Additionally, we show that IL-15 complexes, anti-PD-1mAb, and CD8+ T cells colocalize within the TME. Our results show an increase in CD8+ T cells within the TME, likely due to migrating CD8+ T cells from the tumor-draining lymph nodes. This increase in CD8 T cells within the TME also correlates with increased expression of CD103 (Itgae), a marker associated with tissue residence and improved survival [46]. Our data showing an increase in CD8+ TILs agrees with a recent study using an IL-15 superagonist and anti-PD-L1 fusion protein, N-809, which showed that the influx of CD8+ T cells results from migration rather than in situ proliferation [25,27]. In contrast, another study demonstrated that hetIL-15 administration alters the cytokine and cellular landscape within the tumor, enhancing T cell and cDC1 entry into the tumor and thereby increasing the success rate of immunotherapeutic interventions [64]. Furthermore, we have previously demonstrated that IL-15 complexes can induce migration and increase their cytotoxic capacity without antigen re-stimulation [34]. Moreover, we acknowledge that the timing of IL-15 dosing, either alone or in combination with anti-PD-1, could also affect the antitumor response. We previously showed that CD8 T cell recruitment differs over time after treatment [65]. We hypothesize that CD8 T cells are being heavily recruited from the draining lymph nodes into the tumor 48 h after treatment with IL-15 complexes. In contrast, once the tumors start shrinking, the need for increased numbers of TIL CD8 T cells is no longer required.

Although tumors can exclude immune cells from entering, once inside, tumors create a hostile environment for immune cells, where antitumor effector cells, CD4+ T_H_1 cells, CD8+ T cells, and NK cells are at a competitive disadvantage, given the augmented metabolic activity of tumor cells. One factor that can quickly displace T cells and other effector cells is the low oxygen availability within the TME. Thus, hypoxia is a prominent feature in the TME [66]. HIF-1α is a transcription factor that can sense oxygen levels within the TME and has been reported to positively control PD-L1 expression in myeloid-derived suppressor cells (MDSCs). Moreover, the deletion of HIF-1α has been shown to improve PPAR-α signaling in CD8+ CTLs. Interestingly, our data show that intratumoral treatment with IL-15 complexes combined with anti-PD-1 therapy downregulates hypoxia-related genes, including HIF-1α, and upregulates PPAR-α, consistent with induction of an antitumor response. *Ppargc1α* controls mitochondrial function, oxidative phosphorylation, and reactive oxygen-specific detoxification [67], and enforced expression of PGC-1α in tumor-specific CD8+ T cells has been shown to render them more metabolically active and to enhance cytotoxicity [68]. Furthermore, enforced expression of PGC-1α was shown to promote CD8+ T cell fitness and memory formation [69], critical components of antitumor immunity. The *prkaa2* gene encodes the catalytic subunit of the AMP-activated protein kinase (AMPK). AMPK monitors cellular energy status and increases the accumulation, proliferation, and long-term fitness of effector/memory T cells [70]. We also found that the genes associated with arginine metabolism were upregulated in CD8 T cells. It has previously been shown that arginine metabolism is critical for T cell activation, survival, and driving antitumor activity [41]. While it has been reported that Arginase 1 can be enhanced in tumors, our data would indicate that given the strong bias in the enrichment of genes associated with T cell activation, the lymphoid compartment over the myeloid compartment and overall antitumor responses observed in the double treated groups, combined with the lack of expression in untreated mice, the enhancement in arginase expression is more likely within activated T cells than tumor-associated macrophages and other suppressive immune cells [71].

Integrins play a crucial role in cancer development; however, numerous clinical studies inhibiting their function have failed. We show that integrin expression is differentially regulated by combining IL-15 complexes with PD-1 blockade. Using our combination treatment of IL-15 complexes and anti-PD-1 blockade, which induces tumor immunity, we show that the integrin Itga6 is downregulated. This is in line with a previous report that shows that Itga6 is regulated by hypoxia, and over-expression leads to tumor metastasis and overall tumor progression [51]. Additionally, we demonstrate that combining IL-15 complexes with anti-PD-1 blockade increases integrin expression, favoring a positive antitumor response. These included itgad and itgal, which play a central role in leukocyte adhesion; itgax, a gene encoding CD11c found at high levels on dendritic cells; and itga4, which mediates interactions with the extracellular matrix and cell–cell adhesion.

PVR or CD155 is overexpressed in several human malignancies, whereas its expression is low or absent in most healthy tissues [72,73,74,75,76]. PVR is associated with resistance to anti-PD-1 immunotherapy [77], resulting in immune cell inhibition. In line with previous work, our data show that PVR expression is reduced when IL-15 complexes are combined with anti-PD-1 blockade, thereby, in part, lowering the cancer-immune set-point in the TME to better promote antitumor immunity.

Overall, we found that several genes per gene set show significant fold changes following combination treatment with IL-15 complexes and anti-PD-1 blockade compared to IL-15 complex monotherapy. However, we also acknowledge that some genes showed similarities between IL-15 complexes alone or in combination with anti-PD-1. We predict that the minimal difference between IL-15 monotherapy compared to IL-15 in combination with anti-PD1 in recruiting CD8 T cells and NK cells, along with driving T_H_1 responses, is most likely due to the similarity in the overall immune landscape between the two treatment groups. However, when examining the pathways of these cells within the TME, combination treatment shows trends of greater activity than IL-15 monotherapy, but these differences do not reach statistical significance.

Further experiments are needed to explain the differences in expression of these gene sets between the monotherapy and combinational therapy groups, and to determine how these cumulative differences account for the observed differences in antitumor activity between IL-15 complex monotherapy and combined therapy with PD-1 blockade. We hypothesize that it is most likely that the cumulative effect of these specific gene expression differences associated with combination therapy, compared to IL-15 complex monotherapy, taken together with the significant increases in the many gene scores associated with antitumor activity following combination therapy, necessitates the enhanced tumor control and clearance of EO771 tumors observed with double treatment with IL-15 complexes and anti-PD-1 therapy.

## 4. Materials and Methods

### 4.1. Animal Models

Female C57BL/6 mice, aged 6–8 weeks, were purchased from the National Cancer Institute. Upon arrival, mice were acclimatized for a week under specific pathogen-free conditions with a 12 h light/dark cycle. They had ad libitum access to sterilized food and water. Mice were housed in specific pathogen-free facilities. All experiments were conducted following procedures approved by the Institutional Animal Care and Use Committee (IACUC) and the Institutional Biosafety Committee at Rush University Medical Center.

### 4.2. Tumor Challenge and Treatment with Anti-PD-1 mAb Therapy and IL-15 Complexes

C57BL/6 mice were anesthetized with isoflurane for tumor challenge experiments, and 2.5 × 10^5^ EO771 luminal B tumors (American Type Culture Collection [ATCC], Manassas, VA, USA) were administered in the mammary fat pad (m.f.p). EO771 cancer cell line was cultured in Dulbecco’s Modified Eagle Medium (Gibco, Waltham, MA, USA), 10% fetal bovine serum (Sigma-Aldrich, St. Louis, MO, USA), 100 units/mL penicillin (Gibco), 100 mg/mL streptomycin (Gibco), and 0.29 mg/mL glutamine (Gibco) before harvesting for tumor injection. Once tumors reached 5 mm^2^, mice were treated with either 3.5 μg of IL-15 complex intratumorally (i.t.), 250 µg of anti-PD-1 mAb intraperitoneally (i.p.), or concomitantly with 3.5 µg of IL-15 complexes and 250 µg of anti-PD-1 mAb. For controls, mice were treated with 30 µL of PBS (i.t.) or 250 µg of IgG isotype mAb (i.p.). Four days after initial treatment, one cohort of mice in IL-15 complex treatment groups received a second dose of 3.5 µg of IL-15 complex (i.t.) or 30 µL of PBS (i.t.) for control groups. Mice were treated on a 4-day schedule, receiving 250 µg of anti-PD-1 mAb or IgG isotype control for five treatments. The tumor area was monitored daily via caliper measurement. Mice harboring tumors were killed when the tumor area reached 20 mm in any direction or met other health-related endpoints, per institutional IACUC policies.

### 4.3. IL-15 Complex Treatment and Protein Labeling

Recombinant mouse IL-15 was purchased from eBioscience, and the mouse IL-15Rα-Fc chimeric molecule was purchased from R&D Systems (Minneapolis, MN, USA). IL-15/IL-15Rα-Fc complexes (IL-15 comp) were generated by combining and incubating IL-15 and IL-15Rα-Fc to form a complex in PBS for 30 min at 37 °C as previously described [26]. For immunofluorescent staining and imaging, IL-15Rα-Fc was labeled with Cy5, and anti-PD-1 was labeled with Cy3 via an NHS ester reaction as previously described [71].

### 4.4. Nanostring^®^ PanCancer IO 360^TM^ Panel and GeoMx^®^ Digital Spatial Profiling

We used RNA from the whole tumor treated with PBS or IL-15 complexes with anti-PD-1 mAb and analyzed gene expression using a custom Nanostring^®^ nCounter PanCancer IO 360TM panel (Seattle, WA, USA). This technique uses multiplex hybridization probes bound to a photocleavable linker to target either protein or mRNA, thereby enabling precise quantification of gene and protein expression. Nanostring^®^ GeoMX^®^ digital spatial profiling (DSP) (Seattle, WA, USA), was applied to evaluate spatially resolved RNA profiling in E0771 breast cancer tissue that had been either treated with PBS (i.t.) and IgG isotype mAb (i.p.) or a combination of IL-15 complexes (i.t.) and anti-PD-1 mAb (i.p.). Serial tumor sections from tissue embedded in Optimum Cutting Temperature (OCT) were used to quantify the expression of different cell populations and genes involved in eradicating murine luminal B breast cancer. Sections were shipped to Nanostring^®^ for staining and analysis. Briefly, slides co-incubated with fluorescent-labeled antibodies to detect tumor cells (pan-cytokeratin (CK)), CD8 T lymphocytes, and CD45 for all immune cells, together with DAPI for nuclei detection. The samples were imaged, and at least five regions of interest (ROIs) were analyzed. Nanostring data are representative of two experiments with 3–10 mice per group, unless otherwise stated. A fully annotated gene list in Excel format is available on the Nanostring website for the nCounter PanCancer Mouse IO 360 Panel and the nCounter Mouse Metabolic Pathways Panel.

### 4.5. Quantitative Reverse Transcription-Polymerase Chain Reaction (RT-qPCR)

We collected RNA from the whole mice tumor treated with PBS or IL-15 complex with anti-PD-1 mAb and analyzed the mRNA expression with qRT-PCR select genes to confirm Nanostring^®^ nCounter PanCancer IO360^TM^ panel data. Whole tumor tissue was homogenized using Trizol reagent (Thermo Fisher, Waltham, MA, USA), and RNA was extracted using an RNEasy Mini Kit (Qiagen, Germantown, MD, USA). A SuperScript™ III Platinum™ SYBR™ Green Kit was used for one-step qRT-PCR (Thermo Fisher). The 2(−Delta Delta C(T)) analysis method used CT values from RT-qPCR. The log2 fold change was calculated to represent differential expression between control PBS/IgG isotype-treated and IL-15 complex/anti-PD-1 mAb-treated mice. RT-qPCR data are representative of two experiments with three mice per group.

### 4.6. Cell Score and Counts

The cell type score was calculated as the mean of the log2 expression levels for all the probes included in the final calculation for that specific cell type. Because the scores depend on probe-specific counting and capturing efficiencies, these are interpreted as relative cell abundance values compared to the same cell type within other samples or groups of samples. The scores were not used as measures of the abundance of a cell type relative to different cell types within the same sample or to quantify cell abundance within a single sample. Following background thresholding to negative control genes and normalization to housekeeping genes, gene counts are determined from the number of fluorescent barcodes that hybridize to a specific gene per field of view. For each tumor, gene expression data from the whole tumor is normalized to its expression of housekeeping genes. The fold expression data shown is an average of the expression of specific genes relative to a given treatment (i.e., PBS/IgG isotype). At the same time, pathways scores summarize the frequency of mRNA transcripts of genes associated with a specific pathway for a given sample.

### 4.7. Immunofluorescent Staining and Microscopy

At harvest time, tumors were fresh-frozen, embedded in OCT compound, and stored at −80 °C. Slices were cut on the cryostat in 5 to 10 microns each. The slides were fixed in a solution of 1,4-piperazinediethanesulfonic acid (PIPES) and PFA at a 1:3 ratio, with PFA at 4%. After blocking the tissue with goat serum at a concentration of 10% and BSA at a concentration of 2% for thirty minutes at 25 degrees Celsius, the tissue was stained with primary antibody. Primary antibodies included AlexaFluor^®^ 594 anti-mouse CD103 (Novus Biologicals, Centennial, CO, USA), CD69 (Thermo Fisher^®^), CD8 (Novus Biologicals), and Granzyme B (Invitrogen, Carlsbad, CA, USA). All the primary antibodies were diluted with donkey block serum. Alexa Fluor^®^ 488 rat polyclonal anti-mouse immunoglobulins was used as the secondary antibody for anti-CD8 and anti-CD69 conjugated to Cy5. Both secondary Abs were incubated with the material for one hour at 25 degrees Celsius. Hoechst dye diluted 1:50,000 in PBS was used to stain the tissue nuclei. Before mounting the coverslip, 1–2 drops of the antifade reagent Slow Fade (Thermo Fisher) were applied to each slide. Light-sheet fluorescent microscopy (LSFM) was also performed on tumors and draining lymph nodes harvested 48 h after dual treatment with anti-PD-1-Cy3 and IL-15Rα-Fc-Cy5, labeled via an NHS-ester reaction. LSFM was performed using a LaVision Ultramicroscope (Leica Microsystems, Wetzlar, Germany) at 1.6× magnification and analyzed using FIJI (version 2.0.0) and IMARIS (version 10.1.) software.

### 4.8. Microscopy and Data Analysis

Images were acquired using a Keyence system with a 20×, 40×, or 60× oil objective. To quantify cell singling in tissue, we acquired 10 images at 20× magnification for each type of tissue in the tumor sample. We counted the cells using the Keyence BZ-X800 cell counter (Keyence, Itasca IL, USA).

### 4.9. Statistical Analysis

Results are presented as means with error bars representing s.e.m. Results were compared using an independent *t*-test (also known as a student’s *t*-test), and *p*-values were characterized as follows: * *p* ≤ 0.05, ** *p* ≤ 0.005, *** *p* ≤ 0.0005, **** *p* < 0.0001. Given the small sample size tested, Shapiro–Wilk normality tests were performed to confirm normality of the residuals. Based on these confirmations, subsequent parametric tests were conducted to assess differences in population means. All data measured that passed a normality test with a significance value (alpha) set to 0.05 were then analyzed using ordinary one-way ANOVA and Tukey’s multiple comparison test to determine significant differences between groups. We used Tukey’s *t*-test to assess the significance of the integrated density for immunofluorescent staining.

## 5. Conclusions

In summary, we provide evidence that IL-15 complexes injected intratumorally reach the tumor-draining lymph node, where they can activate and mobilize immune cells, including CD8+ T cells. In addition, we show colocalization of the IL-15 complex/anti-PD-1 within both the tumor-draining lymph node and the TME in the regressing tumor. Our findings indicate that while a single dose of IL-15 complexes administered intratumorally is insufficient in producing a significant change to anti-PD-1 immunotherapy, a second dose generates an increase in anti-tumoral immune markers in murine EO771 breast cancer as well as increased survival. The improved efficacy of intratumoral administration of IL-15 complexes, alone or in combination with anti-PD-1 mAb (i.p.), correlated with increased cytotoxic immune infiltrates, cytokines, chemokines, and co-stimulatory markers. In addition, cytokine and chemokine signaling, metabolism, lysosomal degradation, TCR and co-stimulatory signaling, and cytotoxicity pathways were upregulated. Increased activity of these pathways was directly related to enriched expression levels of individual genes within each pathway. Increasing the number and activity of effector immune cells can boost immunological memory against tumors, with synergistic effects from cytokine-based ICI therapies. Moreover, identifying biomarkers that predict which patients or tumor types are exceptionally responsive to IL-15 superagonist immunotherapy will aid this clinical development. Future studies are needed to determine whether the route and dosing of bifunctional anti-PD-L1/IL-15 superagonists, delivered subcutaneously, or of immunotherapies delivered directly into the tumor can modulate the immune system to convert treatment-refractory patients into responders.

## Data Availability

The original contributions presented in this study are included in the article/Supplementary Materials. Further inquiries can be directed to the corresponding author.

## Supplementary Materials

The following supporting information can be downloaded at: https://www.mdpi.com/article/10.3390/ijms262311490/s1.
