# Supplementary material for: IL-15 Complexes Combined with PD-1 Blockade Affect Immune Cell Distribution, Localization, and Immune Signatures in Regressing Versus Non-Regressing Metastatic Breast Tumors
Source: Int J Mol Sci. 2025 Nov 27;26(23):11490. doi: 10.3390/ijms262311490 (PMC12692021; doi:10.3390/ijms262311490)
Supplement: Supplementary file 1 [file ijms-26-11490-s001.zip › ijms-3823537-supplementary.pdf]

Supplementary Materials

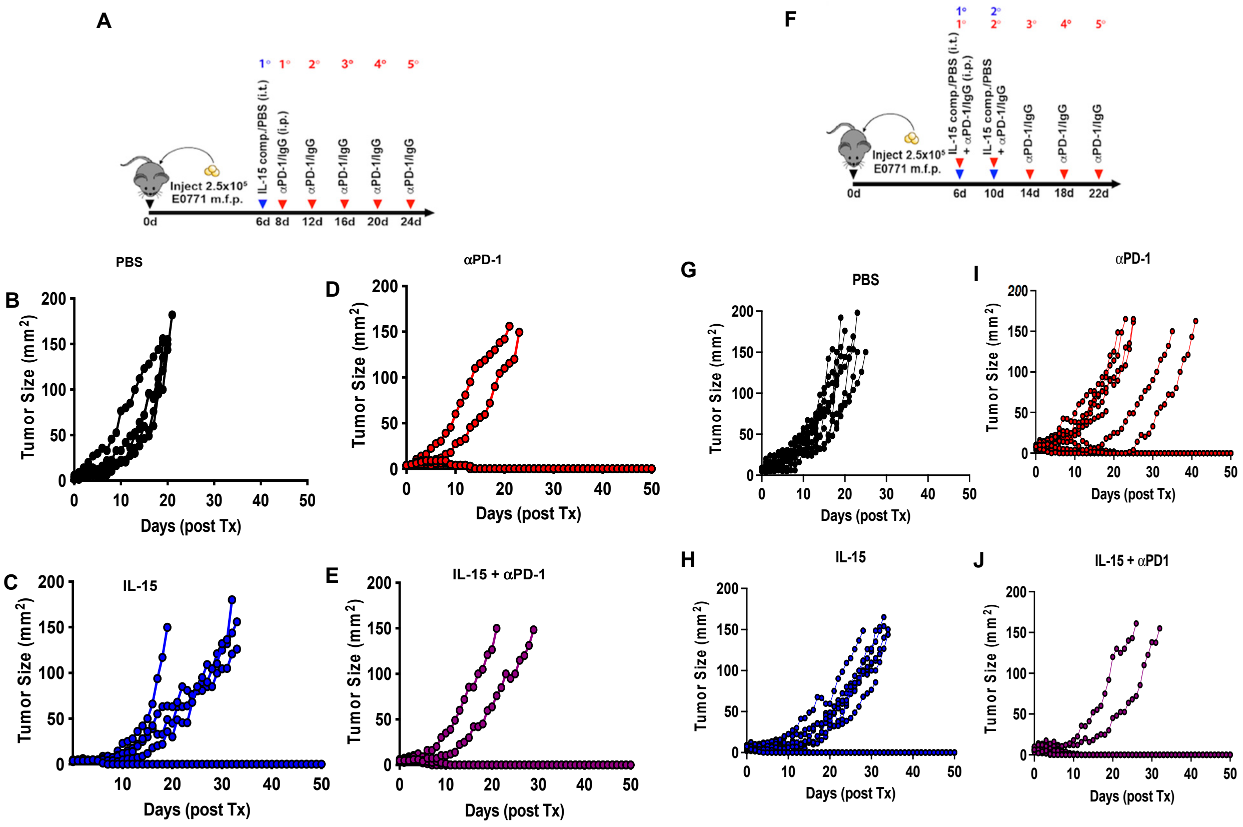

Supplemental Figure S1. Individual tumor progression following intratumoral IL-15 complexes and/or systemic anti-PD-1 mAb therapy. (**A**,**F**) Schematic diagrams indicating the frequency and timing of IL-15 complex and anti-PD-1 administration. (**B**–**J**) Individual tumor progression was measured by tumor area (L × W) and responses in mice treated with PBS (**B**,**G**), IL-15 complex's (**C**,**H**), αPD-1 (**D**,**I**), α-PD-1 (**E**,**J**), and IL-15 complexes (**C**,**H**). Data represent one of three experiments with five mice per group in (**A**–**E**) and twelve mice per group in (**F**–**J**).

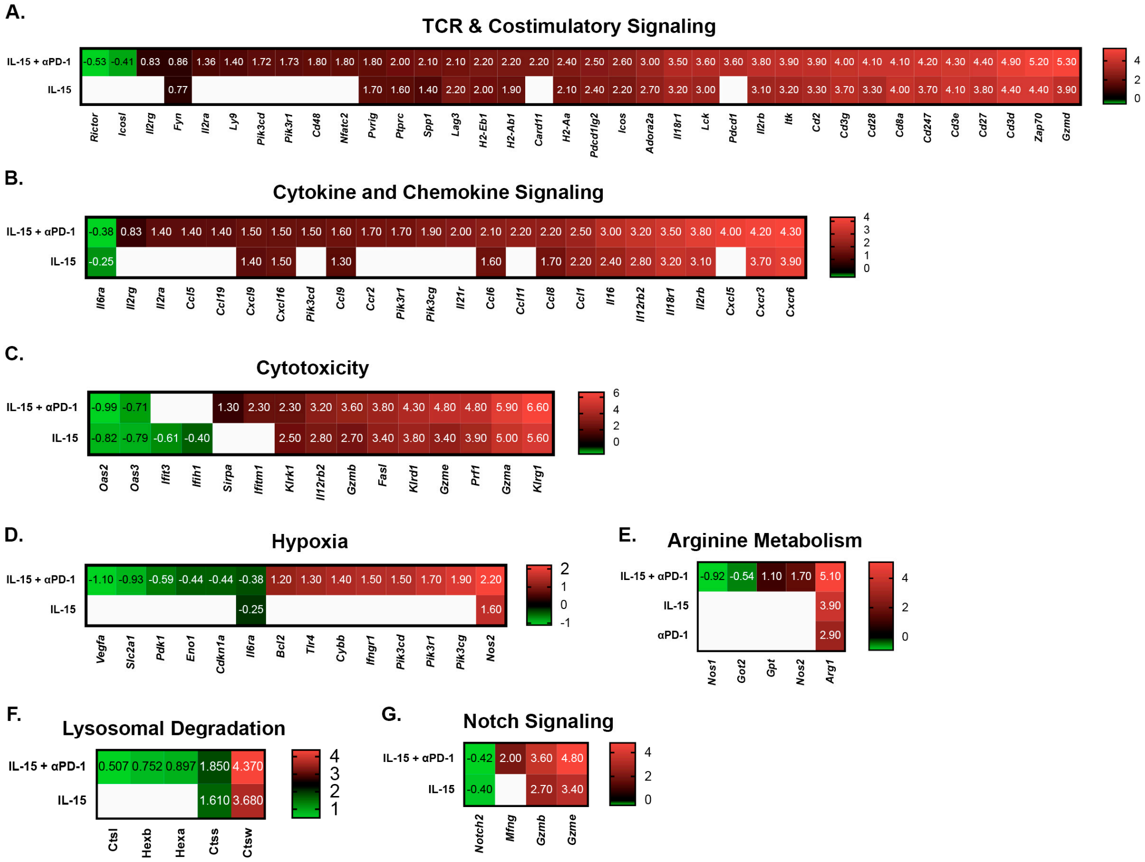

Supplemental Figure S2. Combinatorial treatment with IL-15 complexes and anti-PD-1 therapy induces differential expression of genes involved in immune activation and metabolic pathways. Fold expression changes in genes isolated from total tumor RNA compared to the PBS control treatment group. (**A**) TCR and co-stimulatory signaling heat map. (**B**) Cytokine and chemokine signaling heat map. (**C**) Heat map of cytotoxicity-associated genes. (**D**) Heat map of hypoxia-associated gene score. (**E**) Arginine metabolism heat map. (**F**) Heat map of lysosomal degradation-related genes. (**G**) Heat map of Notch signaling associated genes score. Only Log2 fold change of genes with *p*-values < 0.01 are shown.
